# Adaptive and Maladaptive Correlates of Repetitive Behavior and Restricted Interests in Persons with Down Syndrome and Developmentally-Matched Typical Children: A Two-Year Longitudinal Sequential Design

**DOI:** 10.1371/journal.pone.0093951

**Published:** 2014-04-07

**Authors:** David W. Evans, F. Lee Kleinpeter, Mylissa M. Slane, K. B. Boomer

**Affiliations:** 1 Developmental Neuropsychology Laboratory, Geisinger-Bucknell Autism and Developmental Medicine Institute, Lewisburg, Pennsylvania, United States of America; 2 Department of Neuroscience, Bucknell University, Lewisburg, Pennsylvania, United States of America; 3 Department of Psychology, River Parishes Community College, Sorrento, Louisiana, United States of America; 4 Department of Mathematics, Bucknell University, Lewisburg, Pennsylvania, United States of America; University Children's Hospital Tuebingen, Germany

## Abstract

We examined the course of repetitive behavior and restricted interests (RBRI) in children with and without Down syndrome (DS) over a two-year time period. Forty-two typically-developing children and 43 persons with DS represented two mental age (MA) levels: “younger” 2–4 years; “older” 5–11 years. For typically developing younger children some aspects of RBRI increased from Time 1 to Time 2. In older children, these aspects remained stable or decreased over the two-year period. For participants with DS, RBRI remained stable or increased over time. Time 1 RBRI predicted Time 2 adaptive behavior (measured by the Vineland Scales) in typically developing children, whereas for participants with DS, Time 1 RBRI predicted poor adaptive outcome (Child Behavior Checklist) at Time 2. The results add to the body of literature examining the adaptive and maladaptive nature of repetitive behavior.

## Introduction

Seemingly diverse neurodevelopmental and neuropsychiatric disorders share many features in their respective behavioral phenotypes, and even their possible underlying genotypes [1]. Further, many clinical syndromes are characterized by behaviors that are common in the typical population. Sharp boundaries have historically divided the study of normative and pathological behavior, but developmental psychologists have long noted the benefit of a continuous approach to development and psychopathology [2]–[4]. Such approaches rely on quantitative measures that capture the clinical severity of a given class of behaviors while maintaining sufficient sensitivity so as to examine their distribution in the general population. In this study we examine the similarities and differences in repetitive behaviors and restricted interests (RBRI) in typically developing children and children with Down syndrome (DS). In doing so, we highlight both the normative nature of RBRI, and important differences in the functions and developmental trajectory of RBRI in typically-developing (TD) children and children with a genetically-distinct subtype of intellectual disability – Down syndrome.

The theoretical and empirical attention paid to the study of RBRI pales in comparison to work examining the cognitive, social, and communication deficits associated with neurodevelopmental and neuropsychiatric disorders. RBRI comprise a broad range of behaviors such as compulsions, tics, and stereotypic, self-directed and self-injurious behaviors [5]–[9]. Such behaviors are prominent in a variety of disorders, including autism spectrum disorder (ASD), obsessive-compulsive disorder (OCD), tic disorders, attention deficit/hyperactivity, and a host of other developmental brain dysfunctions [5], [10]–[16].

In a large scale collaborative study on a sample with autism spectrum disorders (ASD) [13] some restricted and repetitive behaviors abated with age, whereas others persisted, indicating that various kinds of repetitive behaviors may have different developmental trajectories. Other work reports that self-injury, insistence on sameness, restricted interests, and compulsions are more frequent among older than younger children with ASD [17], [18]. Such findings illustrate the importance of placing the study of repetitive behaviors in a developmental context – one that transcends chronological age.

RBRI have been examined in several specific genetic copy number variation syndromes. Persons with Prader-Willi syndrome (PWS) for example, engage in compulsive behavior to such a degree that obsessive-compulsive (OC) behaviors may be considered a core aspect of the syndrome [19]. Children with PWS exhibit similar degrees of repetitive and ritualistic behaviors as do children with autism [20], [21] although other work suggests that children with autism exhibit significantly more repetitive behavior, stereotypies, and compulsions relative to children with other developmental disabilities^5^.

Children with DS also engage in restricted behaviors and rituals [15]. A preliminary cross-sectional study revealed that children with DS with mental ages (MA) between 2–5 years engaged in similar levels of RBRI relative to MA-matched typically developing children, differing only in the number of sensory-perceptual symptoms that they exhibited [15], [22]. Older children with DS (MAs >6) engaged in fewer RBRI overall suggesting that children with DS may still “grow out” of their repetitive behaviors, albeit more slowly than typically developing children do [15]. Similar results have been reported on stereotypic motor movements in a heterogeneous sample of children with disabilities, where the development of repetitive motor movements has a delayed trajectory relative to typically-developing children [10]. Unlike the RBRI of typically developing children, however, the RBRI of children with DS was related to other maladaptive behaviors such as internalizing symptoms [15].

A study compared the repetitive behavior questionnaire (RBQ) across several genetic syndromes including Angelman, Cornelia de Lange, Cri-du-Chat, Fragile X, Prader-Willi, Lowe, and Smith-Magenis [23]. Angelman syndrome was associated with relatively few RBRI, whereas Prader-Willi, Cri-du-Chat, and Smith-Magenis syndromes exhibited unique profiles of RBRI [23]. So while RBRI are common across various syndromes, specific genotypes may exhibit differing kinds or degrees of RBRI. The profiles of RBRI are likely to change over development, and different RBRI may be differentially related to adaptive/maladaptive outcomes.

### Normative Repetitive Behavior

RBRI are not limited to neurodevelopmental disorders. Indeed repetitive behavior and restricted interests are part of the behavioral repertoire of many, if not all, typically developing children [7], [9], [16], [24]–[29]. Little is known about the normal development of repetitive behavior; its longitudinal course is not well understood, and we know little about the adaptive and maladaptive correlates of repetitive behavior [14], [30].

In childhood, normal and pathological variants of RBRI may not be easy to distinguish. Typically-developing children exhibit a broad range of rituals, routines, and circumscribed interests that bear an uncanny resemblance to the OC behaviors of OCD and the rigid routines of ASD [16], [24], [25], [28], [30]. Bedtime rituals may include saying goodnight in a certain order to specific people or objects around the house, reading a special book in a certain manner over and over, arranging stuffed animals or other objects in a certain order, and leaving the light on and the door placed ajar with exactness. At mealtimes, the same bowl and cup may have to be used; foods may have to be presented in a certain way, with different foods not touching each other, or sandwiches cut in a certain shape [16], [31], [32].

Cross-sectional research indicates that RBRI are highly prevalent, with as many as 85% of children engaging in RBRI [16]. Preschool-aged children engage in significantly more RBRI than children less than 2 and older than 5 years, although such behaviors remain relatively prevalent throughout the 6^th^ year, and persist to some degree throughout childhood [33], [35]. Children's normative RBRI may be relatively frequent and intense, and may even be associated with some subjective distress [36], making them difficult to distinguish from pathological states.

Although these normative manifestations of RBRI may resemble clinical phenomena, many prominent developmentalists have noted the possible adaptive significance of repetitive, compulsive-like behaviors, such as mastery motivation [31], sensory-motor adaptation in early development [37], and self-regulation [38]. Normative rituals may be linked to magical thinking and may also serve to quell the fears and anxieties that accompany normative development [3]. Empirical efforts support some of these developmental predictions: children's rituals are related to magical beliefs [39], [40] and to normally developing fears and phobias [36]. The development of RBRI is also associated with executive function ability [33], [34], [41], [42] and cortical brain activity [43], suggesting a possible role of neurobiological maturation in the development of RBRI [26], [41], [43], [44].

Normally-developing RBRI have been examined across a variety of cultures using the Childhood Routines Inventory [7], [16], [22], [35], noting high prevalence rates of RBRI in young children in the UK [7], Israel [35], Sweden [21], and Turkey [45]. Animal models provide evidence that repetitive behaviors are common across a variety of species and have both normative and pathological variants that are related to behavioral inhibition and cognitive perseveration [41], [42], [44], [46]–[50].

Common across these research efforts is evidence that RBRI a) are part of normal development; b) are particularly salient early in development (peaking in the preschool years); c) may differentially serve as adaptive or maladaptive functions at different points in development and in different populations. The first goal of this study is to examine the two year course of compulsive-like behavior in typically developing children, and its links to adaptive and maladaptive behaviors. Second, as questions remain as to whether compulsive-like behaviors are part of the behavioral phenotype associated with DS, or whether they reflect a delayed developmental process, we examine changes in RBRI over a two year period in children with and without DS, across two mental age groups. We do so using a quantitative measure of RBRI (the Childhood Routines Inventory [16]) that is sensitive to both the clinical presentation of RBRI, and to the normal distribution of RBRI in typical children. Third, we also examine the adaptive and maladaptive correlates of compulsive-like behaviors in typically-developing children and children with DS; specifically, our goal is to examine whether compulsive-like behaviors early in development predict later adaptive behavior –as would be suggested by a developmental perspective – or whether they reflect prodromal phases of later maladaptive compulsive or autistic-like behavior, and whether such patterns differ for persons with and without DS.

## Methods

### Ethics Statement

This research was approved by the institutional review board of the University of New Orleans. Written consent from all parents and verbal assent from all minors was obtained prior to testing.

### Participants

Participants were two groups of children – with and without DS —tested at two time points separated by 24 months. Each diagnostic group was separated into 2 mental age (MA) groups. Forty-two typically developing children (24 males, 18 females) comprised two MA groups. The majority of the participants were involved in a previous study^15^ on repetitive behavior, which served as Time 1. Seven of the original 50 participants tested at Time 1 relocated, but 2 participants at Time 1 (who were excluded from the original study because they did not meet the age criterion for that study) were included in the present study. At Time 1 the chronological ages ranged from 20 to 86 months (M = 53.76, SD = 20.45), and at Time 2, from 43 to 112 months (M = 77.02, SD = 19.31). The mean difference between age at Time 1 and Time 2 was 23.26 months (SD = 2.55 months). Children's cognitive abilities were assessed with the Stanford-Binet 4^th^ edition. For children less than 30 months of age (at Time 1), the Bayley Scales of Infant Development-II was administered. The mean IQs at Time 1 and Time 2 were nearly identical (Mean at Time 1 = 111.24 (SD = 9.28); Time 2 Mean was 110.76 (SD = 10.39); T1 and T2 IQ *r*(42) = .69, *p*<.001).

The participants were recruited from several sites in an urban region in the Southeastern United States – both public and private schools, and daycare centers. Parents of the participants completed a demographic form including information on parental education, marital status, ethnicity, religion, child's health status, gender, and sibling(s) [if any] as well as sibling's age and gender. The two groups were comparable in terms of the distribution of the demographic variables (all *p*>.40) with the following exceptions: Maternal education of the typical parents was higher than for the parents of DS participants (*p* = .04) and maternal (*p*<.001) and paternal (*p*<.001) ages were higher for the parents of the participants with DS.

For analyses, children were placed into one of two age levels based on Time 1 mental age (CA*IQ/100): We designate as “younger children” those children with MAs 22 to 59 (M = 43.01, SD = 12.76; n = 23) months and “older children” with MAs 60–109 months (M = 79.83, SD = 13.83; n = 19). These age cutoffs were derived from empirical work noting the peak prevalence of RBRI between two and five years of age [16]. The theoretical work of Werner and Gesell highlight the importance of this developmental period as being particularly marked by RBRI.

The group with DS was comprised of 43 persons (18 males, 25 females). These participants were matched on mental age (MA) to the typically developing group at Time 1. The younger group included children with DS whose mental ages ranged between 25 and 59 months (M = 44.71, SD = 10.17; n = 24). Older children ranged in MA from 60.68 to 134 months (M = 74.97, SD = 17.36; n = 19). Chronological ages ranged from 48–253 months at Time 1 (M = 143 months) and from 70–274 (M = 167) months at Time 2. The Time 1 mental ages of the typical children and the children with DS did not differ (*t*(83) = 0.34, *p* = .74). The Typical and DS groups did not differ on the distribution of male and female participants (*χ* ^2^(1) = 1.99, *p* = .159). Participants with DS were also tested with either the Stanford-Binet 4^th^ Ed or the Bayley Scales II: Time 1 IQ, M = 42.02 (*SD* = 7.44) (range = 36–72) and Time 2 IQ, M = 41.70 (SD = 6.49) (range = 36–57); intraclass *r*(43) = .75, *p*<.001(See Table 1).

**Table 1:** Mental Age (MA), IQ, Vineland (VABS) and Child Behavior Checklist (CBCL-Total) Scores at Time 1 and Time 2 for Typically Developing (TD) and DS groups by MA Group.

		MA	IQ	VABS^1^	CBCL^2^	N
TD, Younger						
	(T1)	43(13)	111(9)	102(15)*	52(7)	23
	(T2)	70(14)	111(11)	103(17)	50(9)	23
TD, Older						
	(T1)	80(14)	111(9)	96(13)	50(7)	19
	(T2)	103(14)	110(10)	98(20)	52(9)	19
DS, Younger						
	(T1)	45(10)	44(8)	44(15)	55(7)	24
	(T2)	54(12)	43(7)	37(10)	57(8)	24
DS, Older						
	(T1)	75(17)	39(5)	35(11)	55(7)	19
	(T2)	86(20)	40(6)	33(9)	55(7)	19

Note: Mean values are presented with standard deviations in parentheses.

*N = 20 for T1 VABS Score.

1 Vineland Adaptive Behavior Composite Score; ^2^Child Behavior Checklist Total Problems Score.

### Procedures

Parents received a written description of the proposed study, along with informed consent and several inventories. Willing participants were asked to return the signed consent forms and completed inventories to their child's school, or to the researcher. Children were tested individually at the site of their school, daycare, or home. Parents were then administered a phone interview concerning their child's adaptive behavior level.

### Measures

#### The Childhood Routines Inventory

(CRI) [16] is a 19-item parental report questionnaire that asks parents to indicate the degree to which their child engages in repetitive, ritualistic, behaviors and circumscribed interests along a 5-point scale (frequency/intensity). Unlike other measures of RBRI, the CRI is a quantitative measure that is sensitive to the normal distribution of repetitive behavior in the general population. The CRI has good internal consistency (Cronbach's Alpha = .89), and comprises two factors: “Just Right” phenomena and “Repetitive Behaviors” [16]. The “Just Right” factor includes the items: “prefers to have things done in a particular order or certain way;” “arranges objects or performs certain behaviors until they are just right;” “lines up objects in straight lines or symmetrical patterns;” “insists on having certain belongings around the house in their place;” and “seems very aware of how certain clothes feel.” The repetitive behaviors include: “prefers the same household activities every day;” “acts out the same thing in pretend play;” “engages in elaborate bedtime rituals;” “repeats certain actions over and over;” and “has strong preferences for certain foods” [16]. The CRI yields three indices that reflect mean frequency/intensity scores of the 1–5 Likert scale: the mean Just Right factor (Mean Just Right), the mean Repetitive Behavior factor (Mean Repeat), and the mean frequency/intensity of the overall CRI (Mean CRI). Scale reliability was conducted for the TS and DS samples in the present study. For TD participants Cronbach α = .90; for DS, α = .89.

#### The Child Behavior Checklist

(CBCL) [51] is a 112-item parent-report checklist that assesses child problem behaviors on a 3-point scale. The CBCL has extensive normative data and excellent reliability [51]. The items on the CBCL are factor analyzed into 2 broad-band categories: internalizing (withdrawal/depression) and externalizing (aggressive/delinquent). Two comparable versions of the CBCL were used: one for children less than 4 years old (CBCL/2–3), and the checklist for children 4 years and older (CBCL/4–18). For each scale, raw and standard scores (T scores) are derived.

#### The Vineland Adaptive Behavior Scales

(VABS) Screener [52] is an extension of a widely used measure of adaptive functioning [53]. The VABS Screener, like the VABS survey edition, is a semi-structured interview that assesses the child's current adaptive functioning in four domains: Communication, Daily Living Skills, Socialization, and Motor Skills. The VABS has extensive normative data and excellent psychometric properties. The VABS yields standard scores (M = 100; SD = 15) for each domain, as well as an overall adaptive behavior composite. The screener version of the VABS is correlated highly (*r* = .95) with the full Vineland Survey form [53] and has excellent inter-rater reliability (α = .98).

## Results

The first set of analyses examines the stability of the CRI variables as well as the Vineland and CBCL scales across the two time periods. For each diagnostic group and each MA Level Time 1 and Time 2 correlations are presented. For typically developing younger children, the T1–T2 Mean CRI (*r*(23) = .53, *p* = .009) and Repetitive Behaviors (*r*(23) = .57, *p* = .005) were correlated, but “Just Right” Behaviors were not (although they approached significance at *p* = .051). None of the Vineland scales were correlated across T1 and T2 but both the Internalizing and Externalizing CBCL scales were positively correlated from T1 to T2. (For this MA Level, not all CBCL narrow band scales are directly comparable from T1 to T2 since the CBCL has two slightly different versions for preschool and school age children).

For the older typically developing children, all CRI scores were highly correlated from T1 to T2 (Mean CRI *r* = .79; Just Right *r* = .74; Repetitive *r* = .76, all *p*s<.001). The VABS were correlated across time (Daily Living: *r*(19) = .71, *p* = .001; Socialization: *r*(18) = .46, *p* = .053; Communication: *r*(19) = .77, *p*<.001; Composite: *r*(19) = .80, *p*<.0001. The T1–T2 Internalizing (*r*(19) = .58, *p* = .010) and Externalizing (*r*(19) = .68, *p* = .001) CBCL scales were positively correlated.

For younger children with DS considerable stability was demonstrated for the CRI scales (Mean CRI *r*(24) = .63, *p* = .001; Just Right and Repetitive, *r*(24) = .59, *p* = .002 for both scales). VABS were correlated from T1 to T2 (Composite: *r*(24) = .88, *p*<.0001; Communication (*r*(24) = .87, *p*<.0001; Daily Living (*r*(24) = .71, *p*<.0001; Socialization (*r*(24) = .82, *p*<.0001). The Internalizing CBCL scale was not correlated from T1 to T2, but the Externalizing band was correlated (*r*(24) = .72, *p*<.0001).

Finally, for the older group with DS, all T1–T2 CRI scores were correlated (Mean CRI *r*(19) = .72, *p* = .001; Just Right *r*(19) = .76, *p*<.001; Repetitive *r*(19) = .52, *p* = .022). The VABS Communication (*r*(19) = .52, *p* = .022) and Socialization (*r*(19) = .56, *p* = .012) scores were positively correlated, but the overall Composite and the Daily Living scales were not. The Internalizing (*r*(19) = .65, *p* = .002) and Externalizing (*r*(19) = .72, *p*<.001) CBCL scales were positively correlated over the two time periods.

There is a risk of bias when comparing scores at two separate time points if the variances at these time points are not equal. However, the sample standard deviations for the dependent variables did not differ by more than a factor of 2 (a standard benchmark threshold) across the two time points. Thus we can assume equal variances.

### Linear Mixed Modeling of RBRI

**Figure 1:**
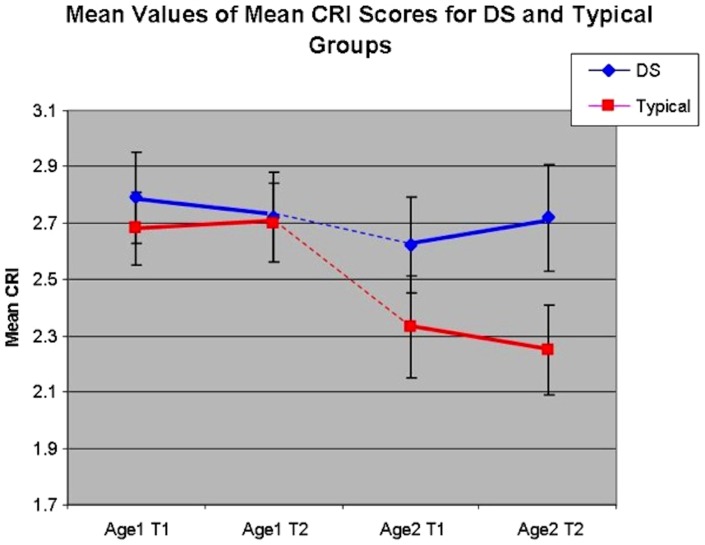
Trajectory of Mean CRI Scores by MA Group and Dx Group.

The main goal of this study was to examine differences in RBRI over time in two age cohorts in participants with and without DS. ID Group by MA level linear mixed multivariate models were performed for each of the three constructs measured by the CRI using a compound, symmetric covariance structure. Levene's tests indicated no significant differences in error variances (all *p*>.05). The first analysis examined the overall mean score of the CRI. Neither the main effects, nor the 2-way or 3-way interactions were significant indicating relative stability in mean CRI scores across the two year span for both children with DS and typically developing children. Thus, mean CRI score trajectories are similar across both ID groups when accounting for MA level. See Figure 1.

**Figure 2:**
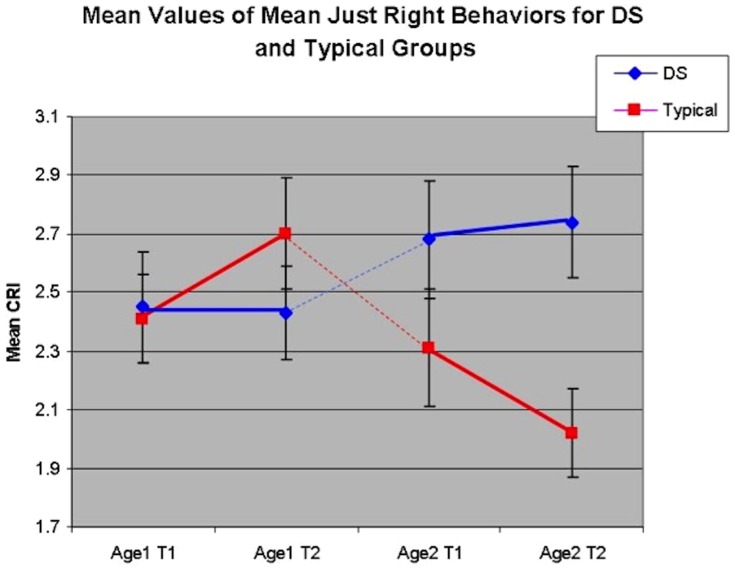
Trajectory of Mean “Just Right” Behaviors by MA Group and Dx Group.

In the mixed model, the “Just Right” factor of the CRI revealed a three-way interaction (Wilks' Lambda *F*(1,81) = 4.26, *p*<.05). Conducted for the separate diagnostic groups, for the typical children, the interaction effect (Age Level X Time) was significant (*F*(1,40) = 5.91, *p*<.05). For the group with DS, this interaction was not significant (*p*>.05), indicating that the overall variance was contributed by changes over time (decreases in Just Right behavior) in the typical children, and relative stability for the children with DS. See Figure 2.

**Figure 3:**
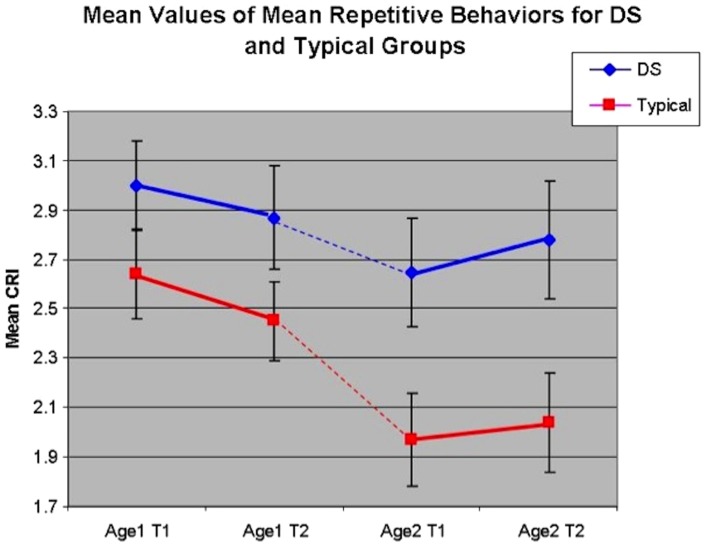
Trajectory of Mean Repetitive Behaviors by MA Group and Dx Group.

Regarding the Repetitive Behavior factor, the 3-way and 2-way interactions were not significant. The main effects for both MA Level (*F*(1,81) = 4.60, *p*<.05) and for ID group (*F*(1,81) = 9.55, *p*<.01) were significant, indicating that Repetitive Behaviors are more frequent/intense in the younger cohort of children than for the older cohort and, overall, differ between typically-developing children and children with DS, such that persons with DS are reported to engage in repetitive behaviors with greater frequency and intensity. When analyzing the diagnostic groups separately, there was a significant main effect of MA group for typically developing children (*F*(1,40) = 5.30, *p*<.05) indicating decreases in RB with increasing age group. No other results were significant. See Figure 3.

### Predicting Adaptive and Maladaptive Behaviors with the CRI

Next, multiple regression analyses tested whether the three CRI indices predict later adaptive and/or maladaptive behaviors. Analyses were conducted within age level and diagnostic groups. For the first series of analyses, each Time 2 standard score from the Vineland Adaptive Behavior Scales was a criterion variable, and each of the three CRI variables from Time 1 served as predictor variables. In this way we examined whether RBRI at Time 1 predict later adaptive behaviors.

None of the CRI variables at Time 1 predicted the overall Vineland Adaptive Behavior Composite score, for either age level or for either diagnostic group. However several significant predictors emerged for the Vineland Domain scores. For older typical children the mean “Just Right” CRI score at Time 1 predicted significant variance in the Vineland Communication Domain at Time 2 (*R* ^2^ = .27, *F*(1,17) = 6.15, *p*<.05). Similarly, Time 1 Repetitive Behaviors was a significant predictor of Time 2 Vineland Daily Living Skills (*R* ^2^ = .27, *F*(1,17) = 6.16, *p*<.05). For older children with DS, Time 1 Mean Repetitive Behaviors predicted Time 2 Vineland Daily Living Skills (*R* ^2^ = .28, *F*(1,17) = 6.50, *p*<.05). The Beta (−.53) reveals that Time 1 repetitive behaviors are inversely related to Time 2 Daily Living Skills, whereas the predictor variables for the typical children were positively related to the Time 2 adaptive criterion variables.

Next, regressions explored whether Time 1 CRI variables predicted later maladaptive behavior as assessed by the CBCL (Internalizing and Externalizing broad band scales). For typically developing younger children, Mean CRI scores at Time 1 predicted significant variance in Time 2 Internalizing scores (*R* ^2^ = .24, *F*(1,21) = 6.68, *p*<.05). For older children with DS, Mean “Just Right” behaviors at Time 1 predicted Internalizing symptoms at Time 2 (*R* ^2^ = .40, *F*(1,17,) = 11.43, *p*<.01.).

To gain a better sense of how early rituals may predict maladaptive behaviors for children with and without DS, we explored the predictive validity of early rituals on T2 narrow band scales of the CBCL (Anxiety, Social Problems, Thought Problems, Attention Problems and Aggression). For younger typically developing children, the Time 1 Mean CRI score predicted significant variance in Time 2 Attention problems (*R* ^2^ = .34, *F*(1,17) = 8.72, *p*<.01). No other CRI variables at Time 1 predicted Time 2 maladaptive behaviors for the typical children. For younger children with DS, the Time 1 Mean CRI score predicted Time 2 anxiety (*R* ^2^ = .25, *F*(1,22) = 7.18, *p*<.05). For older children with DS, the Time 1 mean “Just Right” score predicted significant variance in Time 2 Thought Problems (*R* ^2^ = .26, *F*(1,17) = 6.06, *p*<.05). No other Time 1 CRI scores predicted Time 2 CBCL narrow band scales.

## Discussion

This two-year sequential design examined the development of RBRI and its relation to adaptive and maladaptive behavior in typically developing children and children with Down syndrome. This study extended previous cross-sectional research noting decreases in RBRI in typically developing children [16], and a relative “leveling off” of RBRI in persons with DS [15]. Thus the data elucidate a) our understanding of typical development and possible functions of RBRI; b) the role of cognitive development in the trajectory of RBRI; and c) the ways that RBRI develop as part of the behavioral phenotype of Down syndrome.

As predicted, typically developing children evidence a relatively sharp increase in “Just Right” behaviors throughout the preschool years, followed by significant decreases – both cross-sectionally at the older age levels, and longitudinally (note the significant decrease in “Just Right” behaviors between Time 1 and Time 2 for the older children). For children with Down syndrome, however, “Just Right” behaviors are relatively consistent across the age levels and over time. Children with DS do not experience the normative decreases in frequency/intensity of “Just Right” behaviors that is seen in typically developing children. Indeed, for children with DS, these behaviors persist well throughout childhood and well into adolescence – reaching a delayed asymptote. These findings are consistent with reports of delayed development of other repetitive movements, such as stereotypies and self-injurious behaviors in children with a variety of developmental disabilities [10], [13], [17], [23].

Some repetitive behavior reflects cognitive and behavioral rigidity that precludes more flexible (i.e., adaptive) means of self-regulating and responding to a changing environment [34]. Typical children undergo important developments throughout the preschool and school age years that allow for more flexible and adaptive responses to their environments [54]. As development occurs, new tasks and expectations emerge requiring increasingly flexible and adaptive cognitive and behavioral responses, while maintaining structure and organization. In persons with developmental delays, rituals and routines may persist as the primary means for self-regulation [15], [21]. In typical development, on the other hand, rituals and other compulsive-like behaviors presumably wane with the development of more conceptual and abstract means of self-regulation. Rituals do not disappear with development. Rather, under normal circumstances they become incorporated into relatively more complex and adaptive ways of organizing and regulating the self and the environment [14].

How, then, do early RBRI relate to later development? Some speculate that early developmental rituals may be associated with later behavior problems [28]. An alternative hypothesis suggests that since compulsive-like behaviors are common during early development they likely serve an adaptive function — at least in typically developing children. To date, however, no longitudinal studies have explored these hypotheses.

In the present study, we examined the longitudinal development of RBRI and adaptive/maladaptive behaviors in children with and without Down syndrome. For typically developing children, Time 1“Just Right” behavior predicted later Vineland Adaptive Communication Domain for the older MA level (MA  = 5–11 years). The directionality of the findings suggests that early “Just Right” behaviors augur well for later adaptive behavior in the domain of communication. Similarly, for this same age cohort, early repetitive behaviors significantly predicted Vineland Daily Living Skills two years later. In typically developing children early RBRI are not only statistically normative but also appear to serve some adaptive function later in development. This is rather in contrast to the belief that early RBRI are a risk factor for later symptoms of OCD or ASD, although more research is needed.

For the same MA-matched cohort of children with DS, the Time 1 Repetitive Behavior factor on the CRI also predicted Daily Living Skills two years later. Unlike the finding with typically developing children, the more repetitive behavior a child with DS engaged in at Time 1, the lower his or her Daily Living score two years later. Overall, children with DS in this age cohort engaged in more repetitive behaviors relative to their typical MA-matched cohort. Children with DS may exhibit similar repetitive behaviors in a manner that is perhaps more circumscribed and thus more likely to interfere with, rather than enhance, later adaptive behaviors. This suggests a threshold effect such that, beyond a certain degree, repetitive behaviors are a risk factor for later poor Daily Living Skills. These findings support the ideas put forth by developmentalists such as Gesell [31], [32], Piaget [37], and Werner [3], [57] that for typically developing children repetitive behavior serves as an adaptive mechanism for mastering the environment. Yet early RBRI also predicted maladaptive (i.e., internalizing and externalizing) behaviors in children with and without DS. For the youngest typically developing children, Time 1 CRI scores predicted internalizing behaviors and attention problems two years later. Therefore early rituals and habits seem to predict a combination of adaptive and maladaptive behaviors. Adaptive and maladaptive behaviors are not mutually exclusive. It is quite reasonable to assume that certain kinds of repetitive behaviors may serve some adaptive function while also being linked to anxiety and/or inattention. Such findings may even suggest involvement of neurobiological structures and their development, linking striatal, limbic, and cortico-frontal brain regions that serve as the predominant model of pathogenesis in disorders like OCD [14].

While none of the CRI scores predicted variance in the CBCL broad-band scales for the younger children with DS, the Mean CRI score predicted Time 2 anxiety problems (narrow band). For the older cohort of children with DS, Time 1 Mean “Just Right” Score predicted Time 2 Internalizing symptoms. Regarding the CBCL narrow band factors, the Time 1 Mean CRI “Just Right” score predicted significant variance in Time 2 Thought Problems in the older cohort of children with DS.

In contrast to the findings in typically developing children, where early RBRI appeared to predict a combination of adaptive and maladaptive behaviors later in development, the RBRI of children with DS seem to predict only problem behavior. Children with DS engage in more RBRI than their MA-matched typically developing cohort – particularly at the older MA Level at Time 2 – but these behaviors appear to be more symptomatic. It is possible that repetitive behaviors in persons with DS arise from striatal activation and development, but they are perhaps less organized, integrated and coordinated by frontal-cortical regions, but such interpretation is speculative and merits further investigation.

The results from this study hold some theoretical and practical promise. From a theoretical standpoint, the results speak to continuities and discontinuities in the varieties of repetitive behavior in typically and atypically developing populations. The results may serve as practical information for parents and pediatricians alike who work to distinguish those behaviors that represent the behavioral phenotype of the disability from behaviors that reflect normal developmental processes in persons who are not otherwise experiencing developmental disability [5]. The results of this study suggest that earlier in development, the RBRI of children with DS may be difficult to distinguish from typically developing RBRI and, in this sense, may not be noteworthy. Later in development, RBRI may be considered part of the DS behavioral phenotype rather than a reflection of the typical developmental processes since RBRI predict later problems and disruptions in the acquisition of certain adaptive skills in communication and daily living skills.

The study is not without limitations. Comparing TD children with participants with intellectual disabilities presents special challenges in terms of matching. While matching on developmental constructs such as mental age as we did here is consistent with a developmental approach to disabilities [3], such approaches do not control for experiential or biological factors that may impact behavior in important ways. This approach is unavoidable when comparing individuals with neurodevelopmental disorders to neurotypical groups. Still, future work is needed to evaluate the differential roles of cognitive and biological factors in the development of RBRI. Indeed there is a significant body of work that points to the role of neural structures, such as the basal ganglia, in RBRI [8], [14], [26], [33], [44]. However, there is also evidence linking RBRI with deficits in cognitive control and executive function [14], [26], [34]. So while the repetitive behaviors themselves are strongly influenced by neural structure and function, it is the job of the cognitive systems governing executive functions and self-regulation, more generally, to regulate these behaviors. It is for this reason that we examined groups matched on cognitive level, rather than chronological age.

It is the case that different genetic subtypes of neurodevelopmental disabilities present with varied profiles of RBRI [20], [23] and will likely respond differently to interventions aimed at RBRI to foster better overall adaptive behavior. For this reason, we suggest that future work examines RBRI in specific rare copy number variations associated with NDDs. Other limitations include the use of the Vineland Screener, which though highly correlated with the Survey edition, may yield different results; the necessity of using different measures to assess cognitive ability across age groups may also present bias, although the measures of cognitive development are regarded as comparable.

## Conclusions

For typically developing children, relatively frequent and intense RBRI may be viewed as predictors of later adaptation, albeit with some link to later internalizing symptoms, especially anxiety and inattention. The association between early repetitive behavior and later anxiety/inattention suggests at least some continuity between the normative manifestations of RBRI and those associated with many clinical conditions which may in turn suggest some common underlying neurobiological mechanisms [14], [26].

It is intriguing that some amount of RBRI persists well into childhood when the rigid and circumscribed behaviors are thought to give way to more flexible, adaptive behavior [56], [57]. While RBRI do appear to be more common in children with disabilities and may be considered symptomatic (DS, ASD, PWS [19]–[21]), it is also the case that some repetitive behavior remains part of the repertoire of typical children and adults across cultures [58], [59], possibly representing vestiges of an earlier and once effective means of understanding, organizing, and adapting to a changing environment [14], [55]. This perspective is supported by work examining repetitive behavior in non-human animals as well [41], [49], [60]. Understanding the contextual and organismic factors that determine adaptive and maladaptive manifestations of various kinds of repetitive behavior will serve both theoretical and clinical endeavors.

Understanding the nature and development of RBRI is necessary in order to distinguish those behaviors that may be symptomatic and warranting clinical attention from those that are conspicuous by virtue of their relative asynchrony with other markers of developmental level. Long-term longitudinal studies of both typically developing and non-typically developing children will be necessary to fully understand the developmental nature of repetitive behavior.
